# 1-(2-Amino­eth­yl)-3-phenyl­thio­urea

**DOI:** 10.1107/S1600536811036476

**Published:** 2011-09-14

**Authors:** Pramod Pansuriya, Holger B. Friedrich, Glenn E. M. Maguire

**Affiliations:** a School of Chemistry, University of KwaZulu-Natal, Durban, 4000, South Africa

## Abstract

In the crystal structure of the title compound, C_9_H_13_N_3_S, mol­ecules are linked through N—H⋯S and N—H⋯N hydrogen bonds, forming hydrogen-bonded tapes along the *b* axis. The dihedral angle between the phenyl ring and the thiourea group is 44.9 (2)°.

## Related literature

For the synthesis of the title compund, see: Lee *et al.* (1985 ▶). For applications of thio­ureas, see: Tommasino *et al.* (1999 ▶, 2000 ▶); Leung *et al.* (2008 ▶). For similar structures, see: Guo (2007 ▶); Okino *et al.* (2005 ▶).
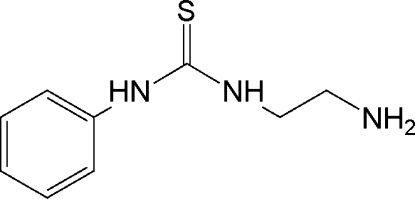


## Experimental

### 

#### Crystal data

C_9_H_13_N_3_S
*M* *_r_* = 195.28
Monoclinic, 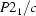

*a* = 8.5105 (2) Å
*b* = 11.5644 (3) Å
*c* = 9.9829 (3) Å
β = 93.580 (1)°
*V* = 980.59 (5) Å^3^
*Z* = 4
Mo *K*α radiation
μ = 0.29 mm^−1^
*T* = 173 K
0.52 × 0.51 × 0.25 mm

#### Data collection

Bruker APEXII CCD diffractometer
11919 measured reflections
2365 independent reflections
1893 reflections with *I* > 2σ(*I*)
*R*_int_ = 0.042

#### Refinement

*R*[*F* ^2^ > 2σ(*F* ^2^)] = 0.033
*wR*(*F* ^2^) = 0.088
*S* = 1.05
2365 reflections
134 parameters
H atoms treated by a mixture of independent and constrained refinement
Δρ_max_ = 0.25 e Å^−3^
Δρ_min_ = −0.31 e Å^−3^

### 

Data collection: *APEX2* (Bruker, 2006 ▶); cell refinement: *SAINT* (Bruker, 2006 ▶); data reduction: *SAINT*; program(s) used to solve structure: *SHELXS97* (Sheldrick, 2008 ▶); program(s) used to refine structure: *SHELXL97* (Sheldrick, 2008 ▶); molecular graphics: *OLEX2* (Dolomanov *et al.*, 2009 ▶); software used to prepare material for publication: *SHELXTL* (Sheldrick, 2008 ▶).

## Supplementary Material

Crystal structure: contains datablock(s) I. DOI: 10.1107/S1600536811036476/fy2013sup1.cif

Structure factors: contains datablock(s) I. DOI: 10.1107/S1600536811036476/fy2013Isup2.hkl

Supplementary material file. DOI: 10.1107/S1600536811036476/fy2013Isup3.cml

Additional supplementary materials:  crystallographic information; 3D view; checkCIF report

## Figures and Tables

**Table 1:** Hydrogen-bond geometry (Å, °)

*D*—H⋯*A*	*D*—H	H⋯*A*	*D*⋯*A*	*D*—H⋯*A*
N1—H1*N*⋯S1^i^	0.898 (17)	2.445 (17)	3.3108 (12)	162.0 (12)
N2—H2*N*⋯N3^ii^	0.836 (15)	2.232 (15)	2.9974 (16)	152.5 (13)

Symmetry codes: (i) 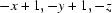; (ii) 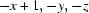.

**Fig. 1.**
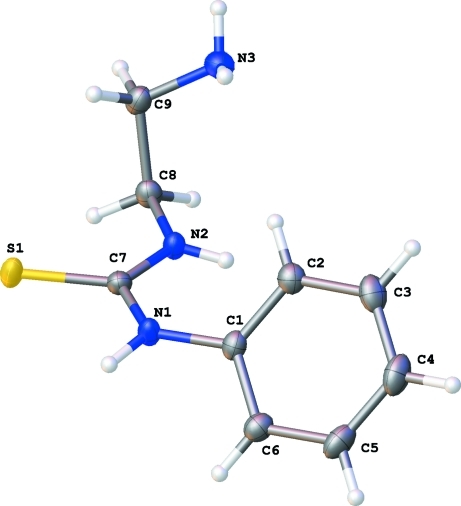
The molecular structure of the title compound. Displacement elipsoids are drawn at 40% probability. Hydrogen atoms are shown as spheres of arbitrary radii.

**Fig. 2.**
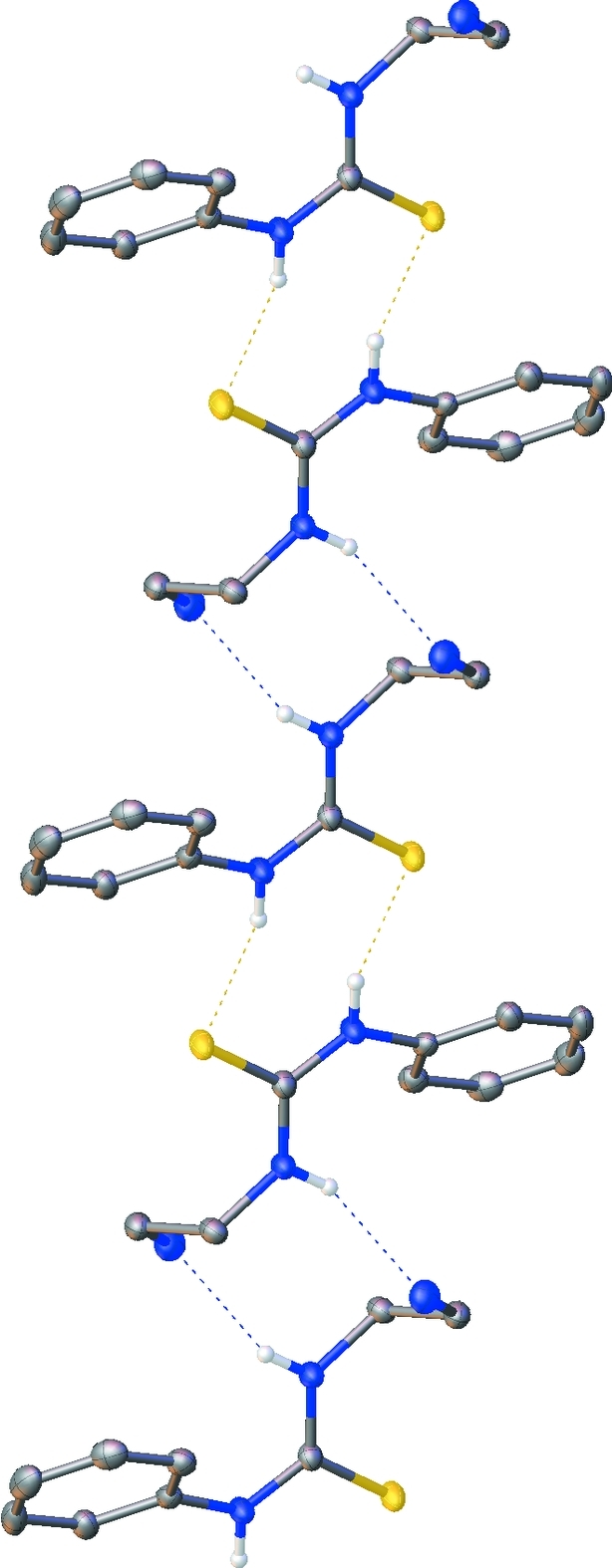
Hydrogen bonding interations viewed on the ab plane. All hydrogens except those involved in bonding have been omitted for clarity.

## supplementary crystallographic information

### Comment

Thioureas have been employed as ligands for metal complexes used in asymetric catalytic hydrogenation (Tommasino *et al.*, 1999, 2000) as well as synthetic anion hosts (Leung *et al.*, 2008). A number of single-crystal X-ray structures have been reported demonstrating a range of inter- and intra molecular hydrogen bonding motifs (Guo, 2007) and (Okino *et al.*, 2005). The title compound is commercially available, but its structure determination (Fig. 1) has not been reported previously.

In the crystal, molecules are linked through intermolecular N—H···S and N—H···N hydrogen bonds (Table 1), forming hydrogen-bonded tapes lying parallel to the *b* axis (Fig. 2). The closest structural analogues all demonstrate intramolecular hydrogen bonding. The hydrogen atoms of the primary amino group are not involved in any short intermolecular contact.

### Experimental

The title compound was synthesized as reported by Lee *et al.* (1985). A solution of phenyl isothiocyanate (6.75 g, 50 mmole) in diethylether (15 ml) was added dropwise at 15°C to a vigorously stirred solution of anhydrous ethylenediamine (6.01 g, 100 mmole) in isopropyl alcohol (100 ml) over a period of 30 min. The reaction mixture was stirred for 2 hrs at room temperature and quenched with water (200 ml). The reaction mixture was maintained overnight at room temperature. Then the reaction mixture was acidified with conc. HCl up to a pH of 2.6. The solvents were evaporated under vacuum and the residue was suspended in hot water for 30 min and the resulting precipitate was filtered. The filtrate was basified by the addition of caustic lye, and a precipitate formed. This in turn was filtered, washed with ice cold water and dried. The yield was 5.06 g. (75%).

Crystals suitable for single-crystal X-ray diffraction were grown by slow evaporation from ethyl acetate at room temperature. M.p. = 408–409 K.

### Refinement

With the exception of those involved in hydrogen bonding, all hydrogen atoms were first located in the difference map then positioned geometrically and allowed to ride on their respective parent atoms with C—H = 0.95 Å and *U_i_*_so_(H) = 1.2*U*_eq_(C) for aromatic and C—H = 0.99 Å and *U_i_*_so_(H) = 1.2*U*_eq_(C) for CH_2_ hydrogen atoms. Hydrogen atoms involved in hydrogen bonding were located in the difference map and refined freely.

### Crystal data

**Table:**

C_9_H_13_N_3_S	*F*(000) = 416
*M**_r_* = 195.28	*D*_x_ = 1.323 Mg m^−^^3^
Monoclinic, *P*2_1_/*c*	Mo *K*α radiation, λ = 0.71073 Å
Hall symbol: -P 2ybc	Cell parameters from 4309 reflections
*a* = 8.5105 (2) Å	θ = 2.4–28.3°
*b* = 11.5644 (3) Å	µ = 0.29 mm^−^^1^
*c* = 9.9829 (3) Å	*T* = 173 K
β = 93.580 (1)°	Plate, colourless
*V* = 980.59 (5) Å^3^	0.52 × 0.51 × 0.25 mm
*Z* = 4	

### Data collection

**Table:**

Bruker APEXII CCD diffractometer	1893 reflections with *I* > 2σ(*I*)
Radiation source: fine-focus sealed tube	*R*_int_ = 0.042
graphite	θ_max_ = 28.0°, θ_min_ = 2.4°
φ and ω scans	*h* = −11→11
11919 measured reflections	*k* = −15→15
2365 independent reflections	*l* = −13→13

### Refinement

**Table:**

Refinement on *F*^2^	Primary atom site location: structure-invariant direct methods
Least-squares matrix: full	Secondary atom site location: difference Fourier map
*R*[*F*^2^ > 2σ(*F*^2^)] = 0.033	Hydrogen site location: inferred from neighbouring sites
*wR*(*F*^2^) = 0.088	H atoms treated by a mixture of independent and constrained refinement
*S* = 1.05	*w* = 1/[σ^2^(*F*_o_^2^) + (0.0456*P*)^2^ + 0.1114*P*] where *P* = (*F*_o_^2^ + 2*F*_c_^2^)/3
2365 reflections	(Δ/σ)_max_ = 0.001
134 parameters	Δρ_max_ = 0.25 e Å^−^^3^
0 restraints	Δρ_min_ = −0.31 e Å^−^^3^

### Special details

**Table:**

Geometry. All e.s.d.'s (except the e.s.d. in the dihedral angle between two l.s. planes) are estimated using the full covariance matrix. The cell e.s.d.'s are taken into account individually in the estimation of e.s.d.'s in distances, angles and torsion angles; correlations between e.s.d.'s in cell parameters are only used when they are defined by crystal symmetry. An approximate (isotropic) treatment of cell e.s.d.'s is used for estimating e.s.d.'s involving l.s. planes.
Refinement. Refinement of *F*^2^ against ALL reflections. The weighted *R*-factor *wR* and goodness of fit *S* are based on *F*^2^, conventional *R*-factors *R* are based on *F*, with *F* set to zero for negative *F*^2^. The threshold expression of *F*^2^ > σ(*F*^2^) is used only for calculating *R*-factors(gt) *etc*. and is not relevant to the choice of reflections for refinement. *R*-factors based on *F*^2^ are statistically about twice as large as those based on *F*, and *R*- factors based on ALL data will be even larger.

### Fractional atomic coordinates and isotropic or equivalent isotropic displacement parameters (Å^2^)

**Table:**

	*x*	*y*	*z*	*U*_iso_*/*U*_eq_	
C1	0.70454 (14)	0.28612 (11)	−0.12947 (13)	0.0231 (3)	
C2	0.67903 (16)	0.18764 (12)	−0.20730 (13)	0.0276 (3)	
H2	0.5781	0.1523	−0.2139	0.033*	
C3	0.80133 (18)	0.14084 (13)	−0.27550 (14)	0.0337 (3)	
H3	0.7842	0.0726	−0.3273	0.040*	
C4	0.94801 (17)	0.19291 (15)	−0.26863 (15)	0.0384 (4)	
H4	1.0311	0.1611	−0.3162	0.046*	
C5	0.97292 (15)	0.29189 (15)	−0.19184 (15)	0.0349 (4)	
H5	1.0734	0.3280	−0.1872	0.042*	
C6	0.85235 (14)	0.33857 (13)	−0.12171 (14)	0.0279 (3)	
H6	0.8704	0.4060	−0.0686	0.033*	
C7	0.46708 (13)	0.29317 (11)	0.00584 (13)	0.0232 (3)	
C8	0.35806 (15)	0.12066 (11)	0.11161 (14)	0.0258 (3)	
H8A	0.3210	0.1745	0.1800	0.031*	
H8B	0.4109	0.0549	0.1593	0.031*	
C9	0.21718 (15)	0.07587 (12)	0.02639 (14)	0.0283 (3)	
H9A	0.1427	0.0379	0.0848	0.034*	
H9B	0.1620	0.1415	−0.0196	0.034*	
N1	0.58075 (12)	0.34090 (10)	−0.06492 (12)	0.0252 (3)	
N2	0.47210 (12)	0.18047 (9)	0.03209 (11)	0.0235 (2)	
S1	0.32346 (4)	0.38159 (3)	0.06009 (4)	0.02980 (13)	
N3	0.26714 (14)	−0.00755 (11)	−0.07427 (13)	0.0323 (3)	
H1N	0.5828 (17)	0.4185 (15)	−0.0621 (15)	0.031 (4)*	
H2N	0.5532 (17)	0.1426 (13)	0.0186 (15)	0.027 (4)*	
H3NA	0.298 (2)	0.0311 (17)	−0.147 (2)	0.056 (6)*	
H3NB	0.186 (2)	−0.0499 (16)	−0.1047 (18)	0.049 (5)*	

### Atomic displacement parameters (Å^2^)

**Table:**

	*U*^11^	*U*^22^	*U*^33^	*U*^12^	*U*^13^	*U*^23^
C1	0.0229 (6)	0.0241 (7)	0.0226 (6)	0.0037 (5)	0.0050 (5)	0.0048 (5)
C2	0.0300 (7)	0.0281 (7)	0.0253 (7)	0.0017 (5)	0.0051 (5)	0.0027 (6)
C3	0.0454 (8)	0.0328 (8)	0.0237 (7)	0.0089 (6)	0.0090 (6)	0.0002 (6)
C4	0.0348 (8)	0.0516 (10)	0.0301 (8)	0.0151 (7)	0.0128 (6)	0.0063 (7)
C5	0.0222 (6)	0.0520 (10)	0.0310 (8)	0.0032 (6)	0.0065 (5)	0.0080 (7)
C6	0.0246 (6)	0.0333 (8)	0.0260 (7)	−0.0006 (5)	0.0036 (5)	0.0032 (6)
C7	0.0203 (6)	0.0219 (7)	0.0275 (7)	−0.0019 (5)	0.0028 (5)	−0.0025 (5)
C8	0.0274 (6)	0.0219 (7)	0.0287 (7)	−0.0015 (5)	0.0075 (5)	0.0018 (5)
C9	0.0235 (6)	0.0244 (7)	0.0373 (8)	0.0010 (5)	0.0047 (5)	0.0031 (6)
N1	0.0227 (5)	0.0176 (6)	0.0362 (7)	0.0001 (4)	0.0095 (5)	−0.0001 (5)
N2	0.0215 (5)	0.0192 (6)	0.0307 (6)	0.0012 (4)	0.0073 (4)	0.0000 (5)
S1	0.02171 (17)	0.0211 (2)	0.0478 (2)	0.00148 (12)	0.01190 (14)	−0.00020 (15)
N3	0.0303 (6)	0.0302 (7)	0.0362 (7)	−0.0015 (5)	−0.0006 (5)	−0.0048 (6)

### Geometric parameters (Å, °)

**Table:**

C1—C2	1.3881 (19)	C7—N1	1.3509 (16)
C1—C6	1.3943 (17)	C7—S1	1.7074 (12)
C1—N1	1.4181 (15)	C8—N2	1.4651 (16)
C2—C3	1.3887 (19)	C8—C9	1.5173 (19)
C2—H2	0.9500	C8—H8A	0.9900
C3—C4	1.384 (2)	C8—H8B	0.9900
C3—H3	0.9500	C9—N3	1.4747 (18)
C4—C5	1.387 (2)	C9—H9A	0.9900
C4—H4	0.9500	C9—H9B	0.9900
C5—C6	1.3871 (18)	N1—H1N	0.898 (17)
C5—H5	0.9500	N2—H2N	0.836 (15)
C6—H6	0.9500	N3—H3NA	0.91 (2)
C7—N2	1.3296 (17)	N3—H3NB	0.882 (19)
			
C2—C1—C6	119.80 (12)	N2—C8—C9	112.61 (11)
C2—C1—N1	121.75 (11)	N2—C8—H8A	109.1
C6—C1—N1	118.27 (12)	C9—C8—H8A	109.1
C1—C2—C3	119.89 (13)	N2—C8—H8B	109.1
C1—C2—H2	120.1	C9—C8—H8B	109.1
C3—C2—H2	120.1	H8A—C8—H8B	107.8
C4—C3—C2	120.50 (14)	N3—C9—C8	110.73 (10)
C4—C3—H3	119.7	N3—C9—H9A	109.5
C2—C3—H3	119.7	C8—C9—H9A	109.5
C3—C4—C5	119.53 (13)	N3—C9—H9B	109.5
C3—C4—H4	120.2	C8—C9—H9B	109.5
C5—C4—H4	120.2	H9A—C9—H9B	108.1
C4—C5—C6	120.53 (13)	C7—N1—C1	129.17 (11)
C4—C5—H5	119.7	C7—N1—H1N	113.9 (9)
C6—C5—H5	119.7	C1—N1—H1N	116.5 (9)
C5—C6—C1	119.73 (14)	C7—N2—C8	123.73 (11)
C5—C6—H6	120.1	C7—N2—H2N	119.9 (10)
C1—C6—H6	120.1	C8—N2—H2N	115.0 (10)
N2—C7—N1	119.23 (11)	C9—N3—H3NA	109.6 (12)
N2—C7—S1	122.64 (9)	C9—N3—H3NB	110.3 (11)
N1—C7—S1	118.12 (10)	H3NA—N3—H3NB	104.6 (17)
			
C6—C1—C2—C3	0.9 (2)	N2—C8—C9—N3	60.48 (15)
N1—C1—C2—C3	176.02 (12)	N2—C7—N1—C1	6.3 (2)
C1—C2—C3—C4	−1.3 (2)	S1—C7—N1—C1	−174.59 (10)
C2—C3—C4—C5	0.7 (2)	C2—C1—N1—C7	44.9 (2)
C3—C4—C5—C6	0.3 (2)	C6—C1—N1—C7	−139.94 (14)
C4—C5—C6—C1	−0.6 (2)	N1—C7—N2—C8	177.90 (12)
C2—C1—C6—C5	0.0 (2)	S1—C7—N2—C8	−1.17 (18)
N1—C1—C6—C5	−175.28 (12)	C9—C8—N2—C7	89.72 (15)

### Hydrogen-bond geometry (Å, °)

**Table:**

*D*—H···*A*	*D*—H	H···*A*	*D*···*A*	*D*—H···*A*
N1—H1N···S1^i^	0.898 (17)	2.445 (17)	3.3108 (12)	162.0 (12)
N2—H2N···N3^ii^	0.836 (15)	2.232 (15)	2.9974 (16)	152.5 (13)

Symmetry codes: (i) −*x*+1, −*y*+1, −*z*; (ii) −*x*+1, −*y*, −*z*.

## References

[bb1] Bruker (2006). *APEX2* and *SAINT* Bruker AXS Inc., Madison, Wisconsin, USA.

[bb2] Dolomanov, O. V., Bourhis, L. J., Gildea, R. J., Howard, J. A. K. & Puschmann, H. (2009). *J. Appl. Cryst.* **42**, 339–341.

[bb3] Guo, H.-M. (2007). *Acta Cryst.* E**63**, o2781.

[bb4] Lee, K. N., Fesus, L., Yancey, S. T., Girardg, J. E. & Chung, S. I. (1985). *J. Biol. Chem.* **260**, 14689–14694. 2865262

[bb5] Leung, A. N., Degenhardt, D. A. & Bühlmann, P. (2008). *Tetrahedron*, **64**, 2530–2536.

[bb6] Okino, T., Hoashi, Y., Furukawa, T., Xu, X. & Takemoto, Y. (2005). *J. Am. Chem. Soc.* **127**, 119–125. 10.1021/ja044370p 15631461

[bb7] Sheldrick, G. M. (2008). *Acta Cryst.* A**64**, 112–122. 10.1107/S0108767307043930 18156677

[bb8] Tommasino, M. L., Thomazeau, C., Touchard, F. & Lemaire, M. (1999). *Tetrahedron Asymmetry* **11**, 1813–1819.

[bb9] Tommasino, M. L., Thomazeau, C., Touchard, F. & Lemaire, M. (2000). *Tetrahedron:Asymmetry* **11**, 4835–4841.

